# The influence of posterior tibial slope on the mid-term clinical effect of medial-pivot knee prosthesis

**DOI:** 10.1186/s13018-021-02704-y

**Published:** 2021-09-15

**Authors:** Weipeng Shi, Yaping Jiang, Xuan Zhao, Haining Zhang, Yingzhen Wang, Tao Li

**Affiliations:** 1 Department of Orthopedic Surgery, The Affiliated Hospital of Qingdao University, No. 59, Haier Road, Qingdao, 266000 China; 2 Medical Department of Qingdao University, Qingdao, 266071 Shandong China; 3 Department of Oral Implantology, The Affiliated Hospital of Qingdao University, Qingdao, 266003 China; 4 Department of Rheumatism and Immunology, The Affiliated Hospital of Qingdao University, Qingdao, 266003 China

**Keywords:** Posterior tibial slope, Medial-pivot prosthesis, Mid-term, Clinical effect

## Abstract

**Objective:**

To evaluate the effect of posterior tibial slope (PTS) on the mid-term clinical outcome following a medial-pivot (MP) prosthesis.

**Method:**

Two hundred thirty-three patients from The Affiliated Hospital of Qingdao University, who had undergone a total knee arthroplasty (TKA) with MP prosthesis between January 2015 and December 2015, were retrospectively included in this study. They were divided into 3 groups according to postoperative PTS: A ≤ 5°; B 5-7°; and C ≥ 7°. Multiple assessments were made on the patient postoperatively and recorded in the three groups, the measurements of this study included: the range of motion (ROM), knee scoring system (KSS), Western Ontario and McMaster universities osteoarthritis index (WOMAC), posterior condylar offset (PCO), joint line height, and postoperative complications.

**Results:**

The average post-operative ROM for groups B and C were 108° and 110° respectively; this was significantly higher than that of group A (98°, *P* < 0.001). The WOMAC scores of patients in group C were significantly lower than those in groups A and B (*P* < 0.05). However, there were no significant differences in KSS, PCO, and joint line height among the 3 groups (*P* > 0.05). Only 2 cases of postoperative complications occurred in group C, these were ameliorated after operation.

**Conclusion:**

With an increase to PTS, the postoperative ROM can be significantly increased for the patient. However, the knee joint function will not be significantly improved, and the stability of knee joint will not be affected when within the limits of appropriate PTS.

**Supplementary Information:**

The online version contains supplementary material available at 10.1186/s13018-021-02704-y.

## Introduction

Knee osteoarthritis (KOA) is a chronic disease involving the synovium and cartilage of the joints. The main purpose of treatment is to relieve pain, improve joint function, and restore range of motion (ROM) to the joint. For patients with mild osteoarthritis, drug and conservative physical treatments are recommended; however, in cases of moderate to severe osteoarthritis, surgical treatment may be proposed as a final treatment option if other treatments prove ineffective [1–3]. Total knee arthroscopy (TKA) is a common surgical intervention, in the USA, more than 600,000 patients receive TKA every year [4]. With the continuous development and improvement of surgical techniques and implants, long-term postoperative patient satisfaction can reach 80% and the survival rate of the prosthesis can exceed 95% [5–7].

Knee range of motion (ROM) is an important factor for the evaluating the effectiveness of a TKA. It is assessed in comparison preoperative ROM, but is taken into consideration alongside multiple factors, these include joint soft tissue condition, surgical approach, surgical technique, prosthetic type, prosthetic location, the height of the joint line, the posterior condylar offset (PCO), and the posterior tibial slope (PTS) [8–12]. The MP prosthesis that was developed in the 1990s opted for a “ball-socket” design. The inner part of the polyethylene gasket was in the shape of a “ball-socket,” to limit the forward and backward movement of the medial femoral condyle. The condyle can still achieve a normal roll-back motion during knee flexion movement; this means the prosthetic mimics a natural knee joint. Not only does this improve knee joint kinematics but it also restores joint stability, joint deep flexion, and reduces wear on the polyethylene gaskets [7, 13].

The published literature suggests that PTS is one of the main factors affecting postoperative ROM. While it is generally believed that increasing PTS can significantly improve the maximum degree of knee flexion after surgery [14–16], the majority of the literature is regarding posterior-stabilized prosthesis (PS) and there is no related research to the use of an MP prosthesis. As each type of prosthesis has significantly different designs, further work needs to be done to assess the effect of PTS on postoperative ROM in patients receiving MP prostheses. In addition, there are physiological and anatomical differences in the knee joints of different races and it has already been demonstrated that these, coupled with gender, may affect joint function following a TKA [17, 18]. It is therefore worth discussing whether the conclusions of previous studies are still applicable to Chinese patients receiving MP prostheses. This study aimed to investigate the effect of PTS on postoperative ROM, joint stability, and function in patients who have received MP prostheses. It is assumed that an increasing PTS will positively correlate with postoperative ROM and that increasing the PTS within an appropriate range will not negatively impact joint stability.

## Materials and methods

Inclusion criteria: (1) Patients diagnosed with knee osteoarthropathy between January and December 2015, who received a TKA in The Affiliated Hospital of Qingdao University; (2) body mass index (BMI) < 35 kg/m^2^; (3) knee varus angle ≤ 20°; (4) perfect preoperative medical records and imaging data. Exclusion criteria: (1) Patients suffering from neurological, psychiatric, and other systemic diseases, unable to cooperate with follow-up; (2) osteoarthritis of the knee combined with immune-mediated comorbidities such as rheumatoid arthritis; (3) a previous history of knee surgery; (4) traumatic arthritis, due to a previous history of knee joint infection; (5) non-operative causes after operation that lead to functional damage of the knee joint.

All operations are performed by the same senior physician team. In this study, MP (Advance Medial-Pivot Knee System, Wright Medical Group) was adopted. All patients were in the supine position; they were given general anesthesia plus a nerve blocking agent. An electric pneumatic hemostatic instrument was used throughout the process, with a pressure of 300 mmHg. A median knee incision was made and the medial parapatellar approach was taken. The synovial membrane was removed, along with a partial fat pad, the meniscus, anterior and posterior cruciate ligaments, and hyperplastic osteophytes. Intramedullary localization was performed on the femur side, and distal eversion of the femur was used for at osteotomy at 5°; extramedullary localization was performed on the tibia side. After the mold test, the patella was repaired. Before the prosthesis was placed, the surrounding soft tissue and posterior joint capsule were subjected to a local infiltration of anesthesia. Finally, tranexamic acid was injected into the joint cavity and a drainage tube was placed. All patients underwent the same postoperative management and rehabilitation program. After the operation, the knee joint was treated with a routine ice compress. Guided ankle pump exercises were performed, and the patient began a multi-mode analgesia regimen opioid painkiller (Tylenol) combined with intravenous flurbiprofen. A low molecular weight heparin anticoagulant was also given and the drainage tube was removed after the drainage volume was less than 50 ml/24 h. Continuous passive machine exercises were carried out on the first day after the operation. Following this, multiple rehabilitation exercises, including leg pressing, lifting, and bending, were performed for 6 weeks following the operation.

**Fig. 1:**
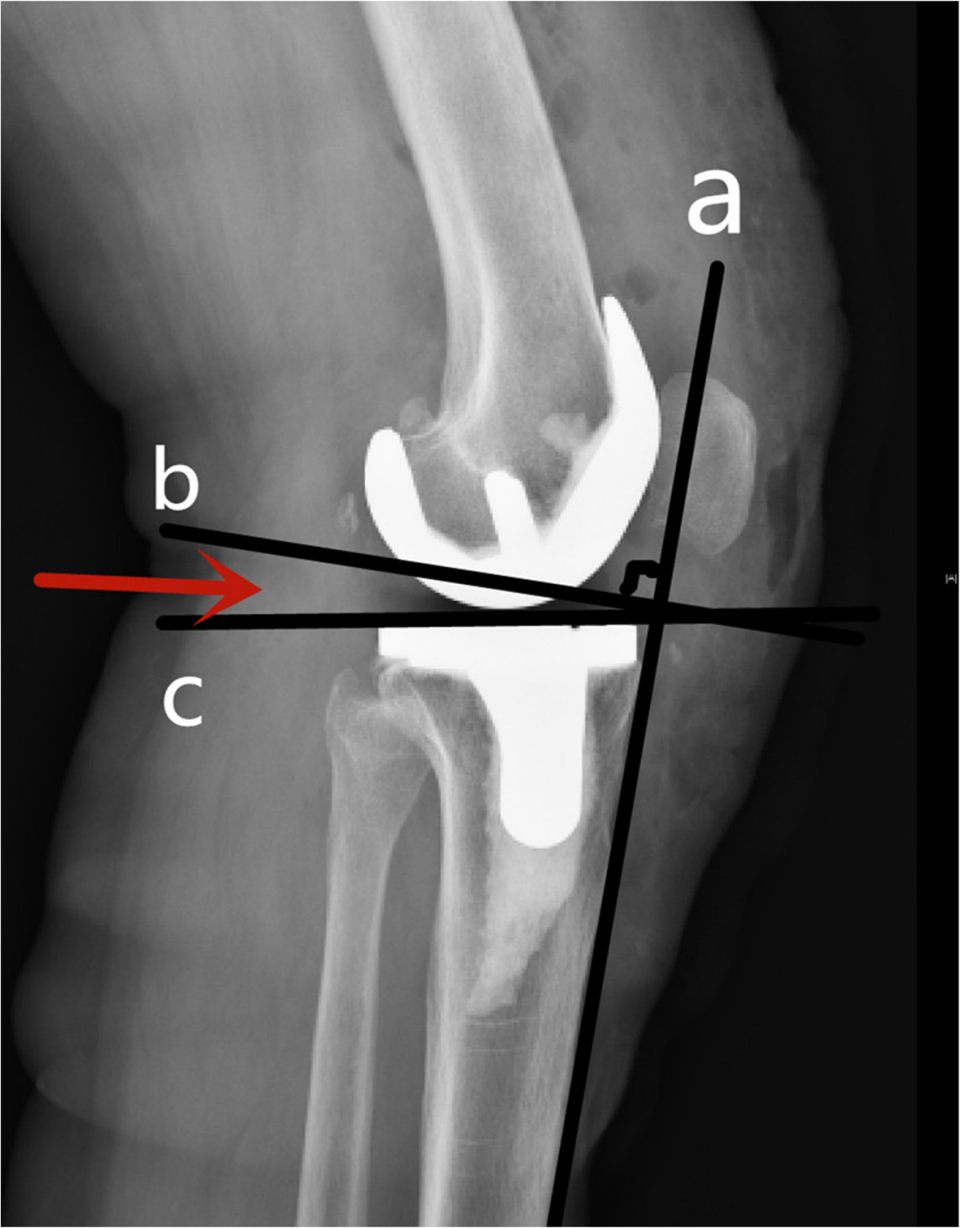
Schematic diagram of a posterior tibial slope. **a** The tibia anterior cortex extension line. **b** Perpendicular to the extension line of the anterior tibial cortex. **c** The connection between the front and rear edges of the tibial plateau. The red arrow displays the angle between the two lines **b** and **c**; this is the posterior tibial slope (PTS)

We defined the complete overlap of the posterior condyle of the femoral prosthesis as a truly accurate lateral X-ray, which was taken by a professional radiologist in pre-operation and on the first day after the operation. The postoperative ROM of all patients was measured by the same experienced joint surgeon using a calibrated goniometer, who was blinded to the patient PTS grouping. In order to reduce the measurement error, the final result was the average of three measurements. The KSS score and WOMAC score were given by two joint surgeons. As the intraoperative tibial osteotomy used extramedullary positioning, the positioning rod was close to parallel with the anterior tibial cortex (ATC) extension line of the upper and middle tibia. Due to this, the measurement standard used the ATC as the reference line measurement. The intersection of the anterior tibial cortex was taken at 15 cm below the knee joint plane and 5 cm below the tibial tubercle, connecting the two points in turn and extending upward as the tangent to the anterior cortex. The point connecting the front and rear edges of the tibial plateau on the lateral view of the knee was the tibial plateau line. The angle between the vertical line of the anterior cortical tangent and the tibial plateau line was the PTS (Fig. 1). PCO used the method proposed by Bellemans [19], by measuring the maximum thickness of the posterior condyle projecting tangentially from the posterior cortex of the femoral shaft. The offset of the posterior condyle before and after the operation was evaluated using X-ray. For the joint line height, the method proposed by Selvarajah [20] was utilized. The joint line was defined as the tangent line passing through the distal end of the femoral condyle, with the joint line height referring to the distance from the proximal end of the fibular head to the joint line. Complications following the surgery include pain in the front of the knee, fractures around the prosthesis, aseptic loosening of the prosthesis, biomechanical instability of the knee joint, and infections around the prosthesis. Two-hundred and thirty-three patients were divided into 3 groups according to postoperative PTS: A ≤ 5° (group A); B 5-7° (group B) and C ≥ 7° (group C).

The collected data was processed using SPSS26.0. Kolmogorov-Smirnov tests were performed to assess the normality of the data. *T* tests were used for normally distributed data and a Mann-Whitney *U* test was used for non-normally distributed data. For enumeration data, the chi-square test was used for comparison between groups and the test level was 0.05.

## Results

### Demography or patient characteristics

A total of 233 patients were included in this study, with an average follow-up time of 52.35 ± 5.82 months. Group A included 68 patients with an average age of 72.31 ± 5.31 years old, and BMI was 27.83 ± 3.65 kg/m^2^; group B included 83 patients with an average age of 71.48 ± 6.12 years old, BMI was 28.11 ± 3.77 kg/m^2^; group C included 82 patients with an average age of 73.29 ± 5.05 years old, and BMI was 28.60 ± 3.44 kg/m^2^ (Table 1).

**Table 1:** Basic information

	Group A (*n* = 68)	Group B (*n* = 83)	Group C (*n* = 82)	Statistics	*P* value
Gender (female, %)	60 (88.2%)	71 (85.5%)	72 (87.8%)	*F* = 0.145	0.865
Age (year)	72.31 ± 5.31	71.48 ± 6.12	73.29 ± 5.05	*Z* = 5.480	0.065
Surgical side (left, %)	26 (31.7%)	44 (52.4%)	40 (48.2%)	*F* = 1.703	0.184
BMI (kg/m^2^)	27.83 ± 3.65	28.11 ± 3.77	28.60 ± 3.44	*Z* = 1.543	0.462

### Preoperative/postoperative PTS and distribution range

**Fig. 2:**
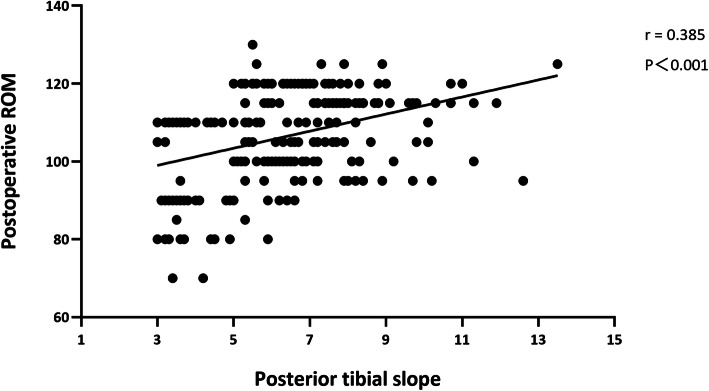
The relationship between the posterior tibial slope and postoperative ROM in all patients. Postoperative ROM increased with increasing PTS, and the two variables were positively correlated (*r* = 0.385)

The average preoperative PTS of all patients was 6.99 ± 1.74° and postoperative PTS was 4.24 ± 2.13°; there was a significant difference between two groups (*t* = 15.295, *P* < 0.001). The distribution of preoperative PTS was relatively concentrated, mainly concentrated between 5.8° and 8.2° [QR, 25~75%], while the distribution of postoperative PTS was relatively scattered, mainly distributed between 2.5° and 5.6° [QR, 25~75%] (Fig. 2).

### Rom

**Fig. 3:**
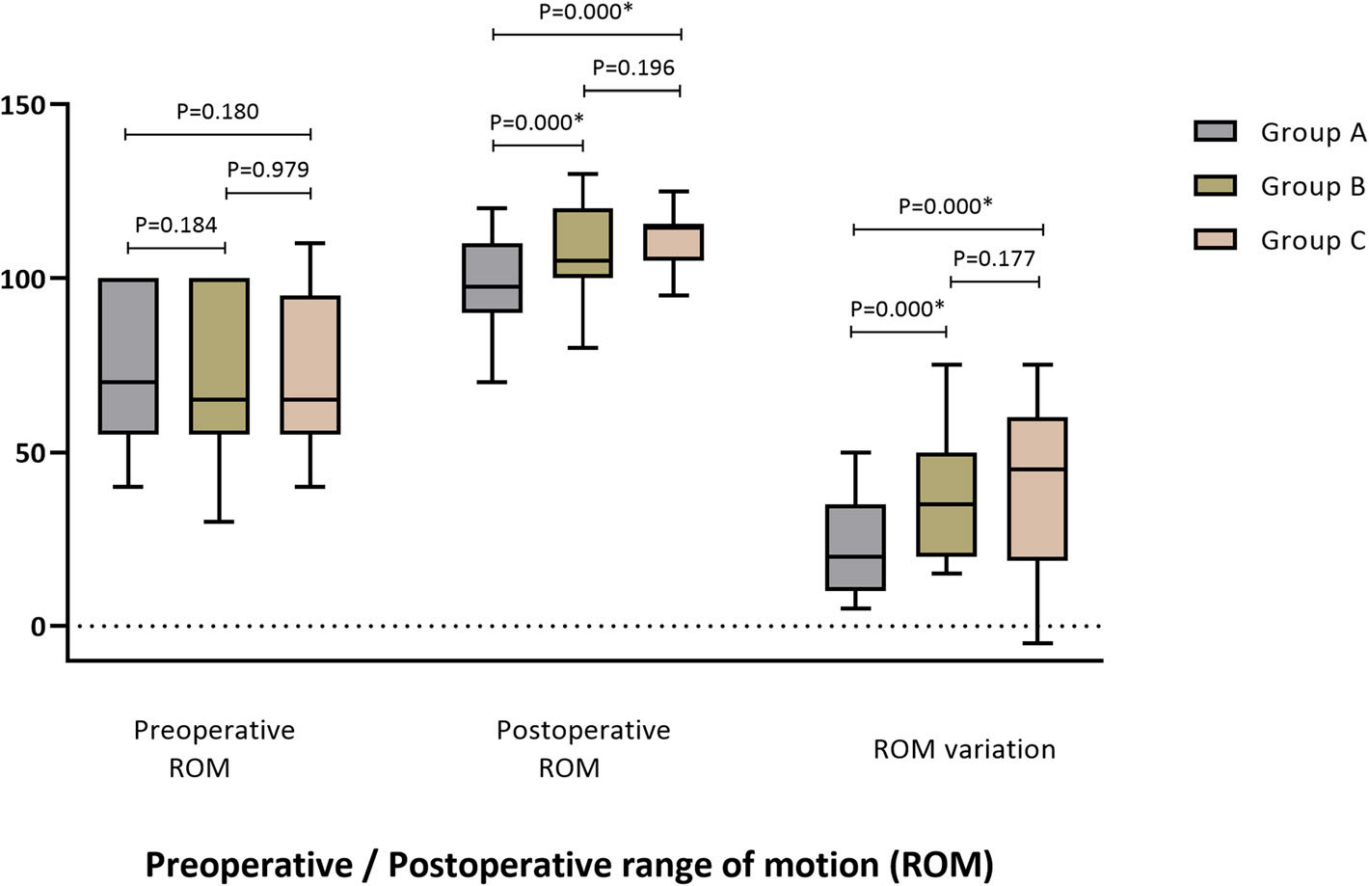
Preoperative/postoperative range of motion (ROM). The postoperative ROM was significantly higher than preoperative ROM in the three groups. There was no significant difference in preoperative ROM between the three groups of patients, and the postoperative ROM of patients in groups B and C were significantly greater than the ROM in group A. In terms of ROM improvement, the effects of surgery on those in group B and group C were better than observed in group A. However, the postoperative ROM of 1 patient in group C was lower than it was before surgery

The average preoperative ROM of patients in groups A, B, and C was 76.03°, 71.87°, and 71.40°, respectively, and there was no significant difference between the three groups (*P* > 0.05). The postoperative ROM of the three groups of patients reached 97.94°, 108.13°, and 110.98°, respectively. Postoperative ROM was positively correlated with PTS (*r* = 0.385, *P* < 0.001). The ROM of groups B and C were significantly higher than that of group A (*P* < 0.001). There was no significant difference between B and C groups (*P* > 0.05) (Figs. 2 and 3).

**Fig. 4:**
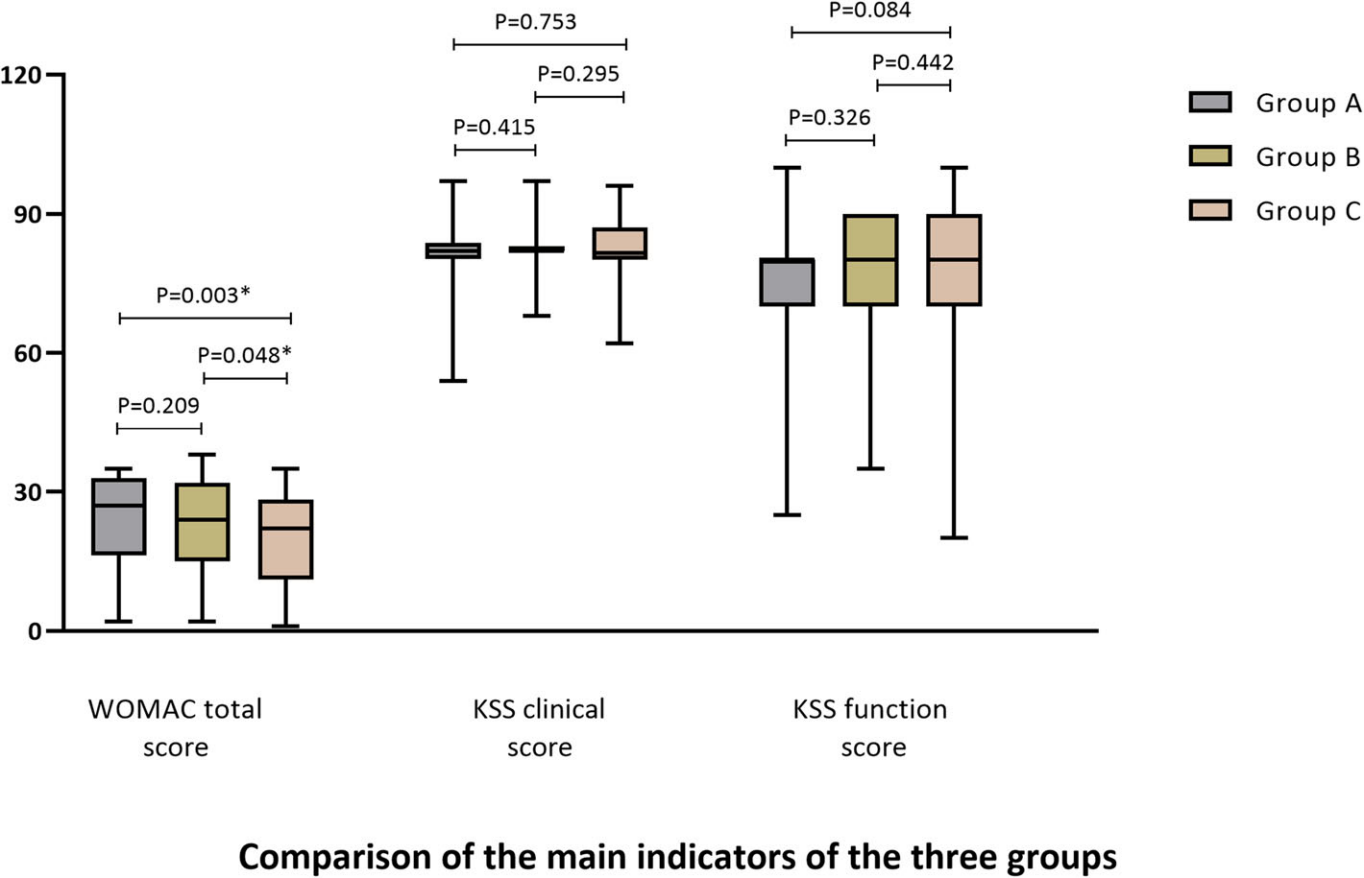
Comparison of the main indicators of the three groups. The postoperative KSS score and WOMAC score of the three groups were significantly improved, but there was no significant difference in KSS and WOMAC between the three groups of patients overall

### Functional score (KSS, WOMAC)

There was no significant difference in the preoperative KSS score and WOMAC score of patients in groups B and C (*P* > 0.05). The clinical and functional scores of the three groups of patients were significantly improved after the operation compared with that before the operation, but there was no significant difference between the groups (*P* > 0.05). WOMAC score, group C had a more significant improvement than groups A and B (*P* < 0.05), and there was no significant difference between groups A and B (*P* > 0.05) (Fig. 4).

### Radiological data (PCO, joint line height)

There was no significant difference in PCO, joint line height, and change the amounts of patients in groups A, B, and C before and after operation (*P* > 0.05) (Table 2).

**Table 2:** Imaging data of three groups of patients

	Group A (*n* = 68)	Group B (*n* = 83)	Group C (*n* = 82)	*Z* value	*P* value
Preoperative PCO (mm)	24.27 ± 3.01	24.39 ± 3.04	24.41 ± 2.76	0.019	0.991
Postoperative PCO (mm)	25.69 ± 2.71	25.87 ± 2.91	26.03 ± 3.25	0.077	0.962
Change of PCO (mm)	1.42 ± 2.13	1.47 ± 2.03	1.62 ± 2.47	0.209	0.901
Preoperative joint line (mm)	16.38 ± 3.76	16.71 ± 3.06	16.56 ± 3.83	0.410	0.815
Postoperative joint line (mm)	17.06 ± 2.97	17.71 ± 2.98	17.20 ± 2.82	1.529	0.465
Change of joint line (mm)	0.68 ± 3.19	1.00 ± 2.86	0.64 ± 3.47	0.996	0.608

### Complication

A total of two postoperative complications occurred in the three groups of patients, all of which occurred in group C. One patient had a periprosthetic joint infection 1 year after surgery, which was *Staphylococcus aureus*, and underwent stage I prosthesis revision surgery to replace the gasket, and the postoperative recovery was good. Another patient developed knee joint pain for half a year after the operation, it was checked that the gasket was prolapsed, and the gasket was replaced by revision surgery, at the last follow-up, there was still slight pain in the anterior area of the knee, and conservative treatment with oral painkillers was utilized, which had no significant impact on daily life. The rest of the patients achieved satisfactory results after the operation, without infection, fracture, aseptic loosening, and other related complications.

## Discussion

In this study, it was identified that in patients receiving an MP prosthesis, PTS was positively correlated with postoperative ROM. With increasing PTS, activity level increased significantly. When the increase to PTS reached a certain angle, the impact on ROM and activity level plateaued. Increasing the level of PTS within an appropriate range was also shown to have no negative impact on joint stability.

Tibial intramedullary localization refers to the placing of the osteotomy guide mold in the tibial medullary cavity; the premise is that the long axis of the medullary cavity coincides with the anatomical axis of the tibia on the coronal and sagittal planes. Although intramedullary localization has the advantages of simple operation, easy assembly, and the positioning process was not susceptible to external interference, it would inevitably damage the medullary cavity structure, increase bleeding, and especially increase the risk of fat embolism. When the tibia is abnormally bent or the medullary cavity is offset, the positioning is not accurate, so the intramedullary localization is greatly restricted in clinical practice. Compared with intramedullary positioning, extramedullary localization has fewer complications. With some surface markers, it can meet the requirements of determining the force line of the lower limbs. It is simple and easy to implement, so it has become the preferred positioning method for most orthopedic surgeons [21].

Up until now, TKA has depended to a certain extent on the operator’s visual judgment and empirical operation to determine tibial osteotomy. Before tibial osteotomy, the positioning function of the tibial extramedullary locator is very important. The tibial extramedullary guide should be parallel to the long axis of the tibia regardless of the frontal view or the side view. However, due to the physiological curvature of the tibia, despite strict extramedullary positioning, the placement of the prosthesis after tibial osteotomy still has a certain degree of deviation, so there is a significant difference between postoperative and preoperative PTS.

A good ROM is essential for normal tasks in daily life, for example, the knee joint of a normal patient needs to flex between 60 and 70°when walking and 90 and 120° when walking up and down stairs or sitting in the chair. As well as this, patients may require an increased ROM for religious activities and movements associated with Asian culture, such as cross-legged sitting and squatting [22, 23]. Although a greater ROM is theoretically more beneficial for the patient in their daily life, excessive postoperative flexology will increase patellofemoral pressure, which may lead to anterior knee pain, excessive wear of the replacement joint, fracture of the patellar, and other complications [24]. Therefore, surgeons generally consider performing an osteotomy to keep the PTS in a suitable range for normal individuals and to restore normal knee kinematics as much as possible.

Some research reported that the angle of the PTS in a normal knee falls roughly in the range of 4-13° [25, 26]. Matsuda [27] found that PTS in the normal knee and arthritic knee joints fell in this range; however, Chiu et al assessed the PTS in Chinese patients with osteoarthritis and it identified as 2 to 3° larger than this “normal” range [28]. Although a small number of studies suggested that increasing the PTS did little to improve the ROM and knee joint function after surgery [29, 30], it was generally thought that a PTS of 10° or lower improves the ROM and does not impact stability. In this study, when the PTS is maintained at 5-7°, there is sufficient range of motion for the posterior femoral condyle when the knee is flexing, resulting in a significantly higher range of motion in this group of patients than in patients with PTS less than 5°. As the PTS of the knee joint continues to bend, the raised posterior lip of the MP prosthesis pad will limit further movement of the posterior femoral cortex, which will in turn limit any improvements to postoperative ROM.

The size of the PTS also relates to the amount of tibial plateau osteotomy. The tibial plateau is an important load bearing structure of the knee joint, the PTS in the normal range allowed the femoral condyle to roll and slide normally in the joint, which ensure good stability and flexion during the extension of the knee joint. When the anteroposterior diameter of the tibial platform is constant, increasing the PTS increases the amount of surface removed in the anterior tibial cortical osteotomy that will expose more fragile bones in the front of the surgical site and lead to a loosening of the tibial prosthesis. Furthermore, a study identified that if the PTS was too small, it may cause too much bone to be removed during the surgery, this can cause increased pressure on the bone, which in turn caused the tibial prosthesis to sink [31]. Performing the cut parallel to the surface of the tibial platform rather than perpendicular to the long axis of the tibial platform provided a 40% higher load bearing capacity, this increases the stiffness by 70% which is enough to support the stability of the tibia prosthesis. The size of the PTS also has a significant impact on the placement space of the tibial component. The osteotomy profile and osteotomy area under different PTS sizes are different, which may further affect the suspension or cause a defect of the prosthesis, limiting its service life.

Joint instability and aseptic loosening are the main reasons for TKA revision. Aseptic loosening is mainly caused by mechanical and biological factors. Interface fretting and stress splintering of the surrounding bone are some mechanical [32]. PTS plays an important factor in reducing the chance of these mechanical stresses. The final interface is one of the main sites of potential wear and interacts with the lateral tibia bone, increasing the size of the PTS increases the coverage of the implant interface. If the PTS is too low, the size of the interface is decreased; this concentrates the stress of movement on a smaller area. This stress can lead to early wearing of the prosthesis, damage to the supporting bone, aseptic loosening of the prosthetic, and eventual failure of the implant. Increasing the interface area between the bone and the implant can improve initial stability of the implant following TKA, the reduction in load bearing stress also reduces the incidence of aseptic loosening and increases the life span of the implant. Therefore, increasing PTS not only improves the postoperative ROM but also may prove to be beneficial in improving postoperative outcomes. In this study, one patient from group C later had follow up surgery to replace the gasket due to recurrent joint pain. It was considered that the PTS was too big in this patient, which led to the buckling clearance being too loose. At the same time, the increased PTS influenced the tension of both sides of the collateral ligament, which caused the tibia and femur articular to loosen gradually. In light of this, it is recommended that increasing PTS to improve knee flexion should be done carefully and stay within the recommended range to avoid joint instability.

Under the same PCO condition, for every 6° increase in PTS, the maximum force on the quadriceps femoris and patellar tendon decreased by 34% [14]. Adding PTS and PCO can enlarge the posterior position of the femoral component, the further back the contact position between the component parts is, the larger the quadriceps lever arm is, thus improving motion efficiency by reducing quadriceps muscle strength required and patella button contact stress, while also reducing extensor device problems [14, 21]. Ostermeier et al. [21] found that in the case of a tibial posterior dip angle of 10°, less quadricep force was required to apply the same stretching moment, especially when the knee flexed above 60°. Kang et al. [14] also concluded through computer simulation that with the increase of PTS, the force of quadriceps femoris and patellar tendon would decrease under any condition of PCO and the force applied to the posterior cruciate ligament would also decrease with a decrease in PTS.

Current research is targeted at PS prostheses, when PS prostheses are involved in moderate bending of the knee, the femoral part of the prosthesis can slide forward and produce contradictory movement. Only when the cam collides with the column will the ideal rollback motion be produced, this column-cam mechanism is relied upon instead of PCL. Due to the possible joint instability during bending and the fact that the curvature of the design can lead to further soft tissue tension and instability, it is suggested that an MP lip be placed before and after the tibial gasket. This provides more stability during bending of the knee joint. During follow-up, we found that for most of the elderly, the priority outcome of the surgery was a reduction in pain and improved stability, while allowing for enough ROM to meet daily requirements. Therefore, we believe that for patients receiving an MP prosthesis, the PTS should be kept between 5-7°. Not only does this achieve an ideal activity level (108°), it avoids the pain and instability problems caused by the large PTS. As Ranawat reported, maintaining a high degree of flexion (140-150°) while being stable and painless is the exception rather than the rule [24].

Although in this study we tried our utmost to match the three groups of patients’ BMI index, PCO, joint line height, preoperative ROM, and used patients who were operated on by the same surgical team, there were still some inevitable factors that may cause error and variability, which was one of the limitations of this study. In addition, due to the wear and degradation of articular cartilage before the surgery, measures of PTS based on the articular surface may not fully reflect the PTS of the knee joint; this may cause errors to the measurement technique.

## Conclusion

Postoperative ROM positively correlated with PTS in Chinese patients receiving MP prosthesis (*r* = 0.385), with a significant increase in ROM when PTS was increased. However, there was no significant difference in knee function under different PTS on the mid-term.

## Supplementary Information

**Additional file 1.**

## Data Availability

All data were contained in the text and charts of published articles.

## Abbreviations

PTS: Posterior tibial slope
MP: Medial-pivot
TKA: Total knee arthroplasty
ROM: Range of motion
KSS: Knee scoring system
WOMAC: Western Ontario and McMaster universities osteoarthritis index
PCO: Posterior condylar offset
KOA: Knee osteoarthritis
PS: Posterior-stabilized
BMI: Body mass index
ATC: Anterior tibial cortex
